# PReS-FINAL-2321: Clinical manifestations of granulomatosis with polyangiitis in 8 children from south-east region of Poland

**DOI:** 10.1186/1546-0096-11-S2-P311

**Published:** 2013-12-05

**Authors:** J Kosałka, M Ignacak, A Zugaj, S Bazan-Socha, K Zachwieja, I Głodzik, G Lis, Z Żuber, J Musiał

**Affiliations:** 1 2nd Department of Internal Medicine, Unit of Allergy and Clinical Immunology, Jagiellonian University, Medical College, Cracow, Poland; 2 2nd Department of Internal Medicine, University Hospital, Cracow, Poland; 3 Pediatric Nephrology, University Children Hospital, Cracow, Poland; 4 Pediatric Pulmonology and Allergology, University Children Hospital, Cracow, Poland; 5 Pediatric Neurology, Rheumatology and Rehabilitation, St. Ludwik Children Hospital, Cracow, Poland

## Introduction

Granulomatosis with polyangiitis (GPA) is a necrotising granulomatous vasculitis affecting the small and medium blood vessels in particular of airways and kidneys. The incidence of GPA in Europe is 25-150 per 1 million per year. This disease typically occurs in the 4th or 5th decades of life and in children it usually cause diagnostic and therapeutic difficulties. Subglottic stenosis and nasal deformity are frequently registered in this group of young patients.

## Objectives

The purpose of this study was to analyse the incidence of GPA in large group of children hospitalized in three paediatric reference centres in south-east administrative region of Poland (3,3 million inhabitants), in the years 1995-2013, as well as to investigate their symptoms, laboratory findings and disease outcome.

## Methods

Retrospective study, examining the medical records. Patients with confirmed diagnosis of GPA must meet criteria of American College of Rheumatology and EULAR/PRINTO/PRES for classification of GPA. All patients were subjected to clinical, laboratory, radiology, immunology assessment.

## Results

We found only 8 children with convinced diagnosis of GPA (6 girls, 2 boys). The average age of onset was approx. 11 years (range: 8-16 years), but the average diagnosis delay was approx. 22 months (range: 0-7 years). The most common clinical features at presentation were constitutional symptoms - weight loss, fever and arthralgia (87.5% - 7/8). The frequency of system involvement at presentation was: kidneys 87.5% (7/8), lungs 75% (6/8), ear/nose/sinuses/throat 50% (4/8), gastrointestinal tract 50% (4/8), skin 37.5% (3/8), eyes 12.5% (1/8), joints 12.5% (1/8) and nervous system 12.5% (1/8). ANCA were positive in all patients. Treatment included: glucocorticosteroids 100%, cyclophosphamide 100%, mycophenolate mofetil 50%, plasmaferesis 37.5%, hemodialysis 25% and in 12.5% cyclosporine. 4 children has or had progression of the disease, in spite of appropriate treatment (1 has constant progression of sinusitis, 2 has end-stage renal failure, 1 died because of alveolar haemorrhage).

## Conclusion

Female predominance and clinical features of GPA diagnosed in children were similar to those described in adults. However, none of our patients had subglottic stenosis and only in 2 cases was observed saddle-nose. Although GPA was appropriate treated, progression was observed in 50% children.

## Disclosure of interest

None declared.

